# Leveraging the ATP‐P2X7 receptor signalling axis to alleviate traumatic CNS damage and related complications

**DOI:** 10.1002/med.21952

**Published:** 2023-03-16

**Authors:** Yaling Yin, Linyu Wei, Emily A. Caseley, Osbaldo Lopez‐Charcas, Yingjuan Wei, Dongliang Li, Steve P. Muench, Sebastian Roger, Lu Wang, Lin‐Hua Jiang

**Affiliations:** ^1^ Sino‐UK Joint Laboratory of Brain Function and Injury of Henan Province, Department of Physiology and Pathophysiology Xinxiang Medical University Xinxiang China; ^2^ Faculty of Biological Sciences, School of Biomedical Sciences University of Leeds Leeds UK; ^3^ EA4245, Transplantation, Immunology and Inflammation, Faculty of Medicine University of Tours Tours France; ^4^ Sanquan College of Xinxiang Medical University Xinxiang China

**Keywords:** CNS‐penetrant P2X7 receptor antagonist, extracellular ATP, microglial activation, neuroinflammation, P2X7 receptor, proinflammatory cytokine, therapeutics, traumatic CNS damage

## Abstract

The P2X7 receptor is an exceptional member of the P2X purinergic receptor family, with its activation requiring high concentrations of extracellular adenosine 5ʹ‐triphosphate (ATP) that are often associated with tissue damage and inflammation. In the central nervous system (CNS), it is highly expressed in glial cells, particularly in microglia. In this review, we discuss the role and mechanisms of the P2X7 receptor in mediating neuroinflammation and other pathogenic events in a variety of traumatic CNS damage conditions, which lead to loss of neurological and cognitive functions. We raise the perspective on the steady progress in developing CNS‐penetrant P2X7 receptor‐specific antagonists that leverage the ATP‐P2X7 receptor signaling axis as a potential therapeutic strategy to alleviate traumatic CNS damage and related complications.

## 1 INTRODUCTION

The concept of purinergic signaling originated from the landmark discovery, made by Geoffrey Burnstock half a century ago, of adenosine 5ʹ‐triphosphate (ATP) as a neurotransmitter released by the so‐called nonadrenergic, noncholinergic nerve terminals in the gut and bladder.^1^, ^2^ As is well recognized nowadays, ATP is released from many types of mammalian cells as an autocrine and/or paracrine signaling molecule in response to a diversity of physical, chemical, and biological stimuli.^3^, ^4^, ^5^, ^6^, ^7^, ^8^, ^9^ Decades of research efforts have established the P2 purinergic receptors as intrinsic mechanisms that mediate the miscellaneous actions of extracellular ATP. P2 receptors are categorized into two functionally and structurally distinctive subfamilies; ligand‐gated ion channel P2X receptors and G‐protein‐coupled P2Y receptors. Mammalian cells express seven genes encoding P2X receptor proteins (P2X1–P2X7) and eight genes for P2Y receptors (P2Y1, P2Y2, P2Y4, P2Y6, P2Y11–P2Y14).^10^

P2X receptors are homo/hetero‐trimers and function as an ion channel permeating K^+^, Na^+^, and Ca^2+^.^11^, ^12^, ^13^, ^14^, ^15^ The homo‐trimeric P2X7 receptor displays a strikingly low sensitivity to ATP, with its activation requiring concentrations one or two orders of magnitude higher than for activation of other P2X receptors.^16^ In addition, sustained activation of the P2X7 receptor triggers the formation of large pores across the plasma membrane that allow passage of organic cations that are considerably larger in size than physiological cations.^17^, ^18^, ^19^ These exceptional functional properties of the receptor, initially observed in macrophages, earned the name of P2Z receptor to distinguish it from the canonical ligand‐gated ion channel P2X receptors.^20^ Subsequent molecular cloning predicted it to have the same membrane topology as the P2X receptors, leading to its redesignation as P2X7, the last member of the P2X receptor subfamily as it is known today.^21^, ^22^, ^23^ The P2X7 receptor is expressed in a variety of tissues and cells, and participates in a plethora of physiological and pathological processes.^24^, ^25^, ^26^, ^27^, ^28^, ^29^, ^30^, ^31^, ^32^ As an extensively researched example, the P2X7 receptor in immune cells plays a crucial role in mediating immunity and, not surprisingly, numerous inflammatory diseases.^33^, ^34^, ^35^, ^36^ Recently, it has attracted mounting attention as a potential culprit in the induction of the “cytokine storm” responsible for severe pneumonia and neuropathology due to COVID‐19.^37^, ^38^

The central nervous system (CNS), including the spinal cord and brain, controls most of the body functions through coordinated actions of neurons. There also exist a large number of nonneuronal or glial cells, with microglia, astrocytes, and oligodendrocytes being the major types in the mature CNS, and they are vital in maintaining CNS homeostasis and function.^39^, ^40^, ^41^, ^42^ Microglia are the major immunocompetent cells, often referred to as CNS‐resident macrophages despite increasing evidence indicating significant differences in their origin and morphological and functional phenotypes.^42^ Under normal conditions, microglia adopt a ramified morphology characteristic of a small cell body with multiple fine processes, and patrol the CNS parenchyma with the extensive networks of their fine processes. Microglia support neuronal function by secreting neurotrophic factors and removing unwanted synapses and cells as well as neurotoxic molecules via phagocytosis. Furthermore, microglia can produce a tightly regulated cascade of pro‐ and anti‐inflammatory effects on the CNS. Microglia express a battery of pattern recognition receptors (PRRs) for danger‐associated molecular patterns (DAMPs) released from host cells, with ATP being one, and pathogen‐associated molecular patterns (PAMPs) from invading pathogens. Upon activation, microglia assume an ameba‐like morphology and initiate a proinflammatory response to clear tissue damage or infection, followed by an anti‐inflammatory response to repair tissue damage and terminate the immune response. However, aberrant microglial activation, or chronic microglial activation even at a low level, can result in undesirable generation of proinflammatory mediators that damage healthy neural tissues leading to loss of neurological and cognitive functions, a process termed neuroinflammation. A large volume of evidence supports that microglia‐mediated neuroinflammation is a common and major mediator of a variety of CNS diseases.^40^, ^41^, ^42^, ^43^, ^44^, ^45^, ^46^, ^47^, ^48^, ^49^ Thus, it is imperative to gain a clear understanding of the mechanisms driving microglial activation and neuroinflammation for the development of therapeutic strategies restoring microglial functions and treating CNS diseases.

The P2X7 receptor was in fact first cloned from a rat brain tissue cDNA library,^21^ and its expression and role in the CNS have been extensively researched. The expression of the P2X7 receptor in glial cells is widely accepted, whereas its expression in neurons has been a topic of heated debate,^50^, ^51^, ^52^ which remains unsettled.^53^, ^54^, ^55^ The P2X7 receptor serves an important mechanism mediating the communications between neurons and glia.^56^ The P2X7 receptor in microglia also acts as the PRR for ATP released from degenerating and stressed cells, and the ATP‐P2X7 receptor signaling axis plays a critical role in microglial activation and neuroinflammation in the pathogenesis and development of multiple neurodegenerative diseases and psychological disorders. Readers interested in such topics can consult recent reviews.^27^, ^56^, ^57^, ^58^, ^59^, ^60^, ^61^, ^62^, ^63^, ^64^ Accumulating evidence also shows that ATP release, microglial activation, and neuroinflammation are common events leading to loss of neurological and cognitive function following a variety of traumatic CNS damage conditions, including spinal cord injury (SCI), traumatic brain injury (TBI), ischemic stroke (IS) and hemorrhagic stroke, neonatal hypoxia‐ischemia (NHI), radiation‐induced brain injury (RBI), and perioperative neurocognitive disorder (PND). In this review, we discuss the ATP‐P2X7 receptor signaling axis, drawing attention to its role in microglia‐mediated neuroinflammation in CNS damage resulting from these conditions. We start with a brief introduction of the recent breakthroughs in delineating the structural basis for P2X7 receptor activation by ATP. We discuss the studies investigating the role of the P2X7 receptor and underlying mechanisms in traumatic CNS damage conditions. Finally, we raise the perspective on CNS‐penetrant P2X7 receptor antagonists as potential therapeutics to alleviate the aforementioned traumatic CNS damage conditions and related complications.

## 2 STRUCTURAL BASIS FOR ATP BINDING AND RECEPTOR ACTIVATION AT THE P2X7 RECEPTOR

**Figure 1:**
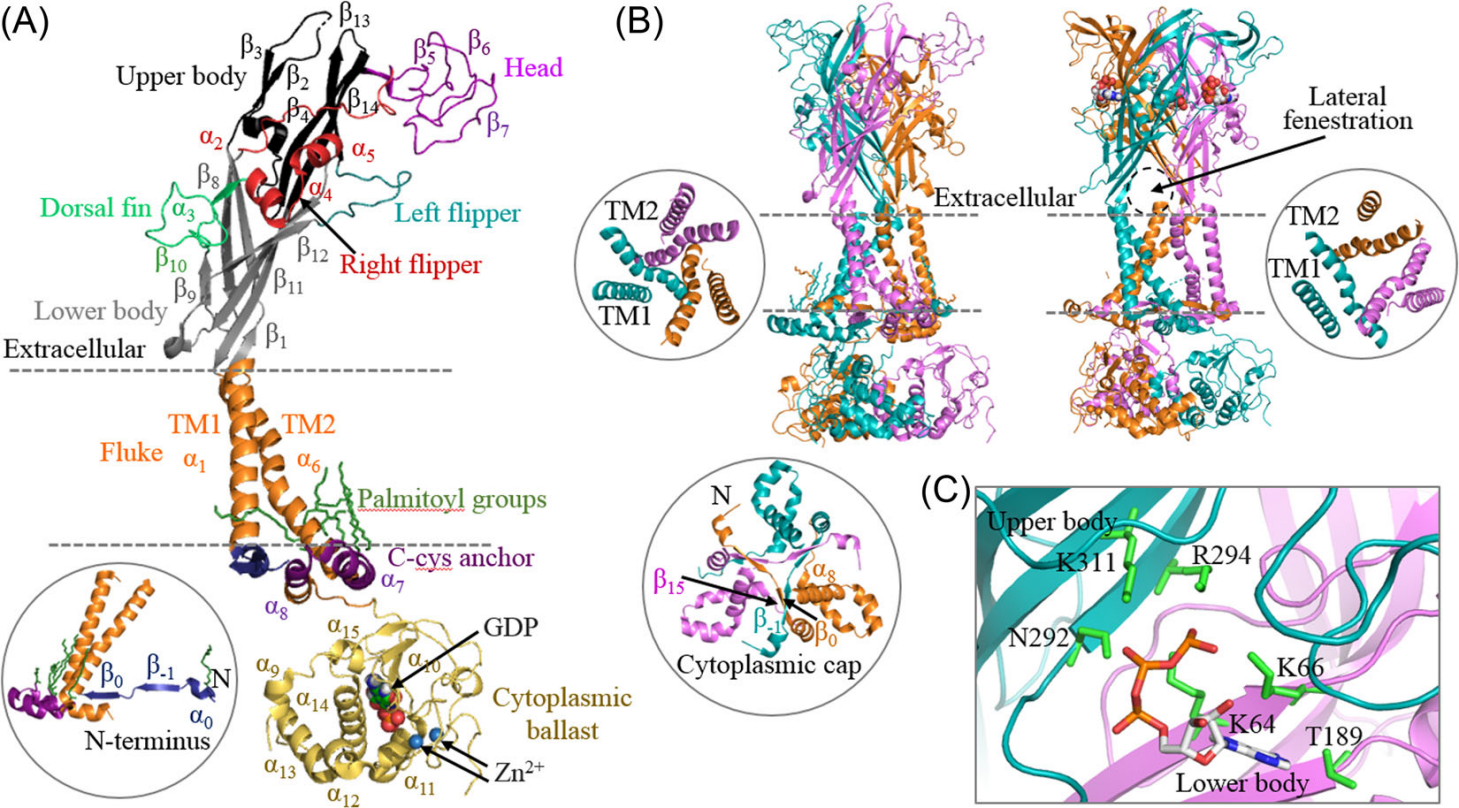
Structural features of the P2X7 receptor. (A) The structural features of the rat P2X7 receptor protomer or subunit. Insert: the N‐terminus. (B) The architectures of the homo‐trimeric receptors in the apo, closed state (left) and ATP‐bound, open state (right), with the three subunits indicated in different colors. Insets: the ion pore in the closed state (left) and open state (right), viewed from intracellular side, and the cytoplasmic cap and cytoplasmic ballast (below). (C) The six conserved residues in the rat P2X7 receptor that coordinate ATP binding to the intersubunit site. ATP, adenosine 5ʹ‐triphosphate. [Color figure can be viewed at wileyonlinelibrary.com]

Each subunit or protomer of the trimeric P2X receptor is composed of two transmembrane domains (TM1 and TM2), a large extracellular domain, a short intracellular N‐terminus, and an intracellular C‐terminus of variable length.^11^ As illustrated in Figure 1A for the structure of the rat P2X7 receptor in the apo, closed state, both transmembrane domains are α‐helical (TM1/α_1_ and TM2/α_6_), and the extracellular domain contains 4 α‐helices (α_2_–α_5_) and 14 β‐strands (β_1_–β_14_),^65^ which are similar to the other P2X receptors.^66^, ^67^, ^68^, ^69^, ^70^ The N‐terminus of the P2X7 receptor has one α‐helix (α_0_) and two β‐strands (β_−1_ and β_0_), whereas its exceptionally long C‐terminus encompasses 10 α‐helices (α_7_–α_16_) and 4 β‐strands (β_15_–β_18_) (Figure 1A). The recently determined structures of the rat P2X7 receptor consist of three parts, extracellular, transmembrane, and intracellular (Figure 1B). The three extracellular domains intertwine tightly with one another to form the extracellular part. In the transmembrane part, the three TM2 domains line the central ion‐permeating pore and the three TM1 domains position at the periphery of the receptor (Figure 1B). The intracellular part of the P2X7 receptor is made of three distinctive structural modules, named the cytoplasmic cap, C‐cys anchor, and cytoplasmic ballast.^65^ The cytoplasmic cap is formed by β_15_ and α_8_ in the proximal C‐terminus, together with two N‐terminal β‐strands from two adjacent subunits, β_−1_ from one subunit and β_0_ from another subunit (Figure 1B). The C‐cys anchor and cytoplasmic ballast are P2X7 receptor‐specific. The C‐cys anchor denotes the cysteine‐rich domain in the proximal C‐terminus that connects the TM2 and cytoplasmic cap, and tether them to the plasma membrane, thanks to its extensive palmitoylation at multiple cysteine residues (Figure 1A). Such an arrangement stabilizes the conformation of the ion channel in the open state,^65^ providing the structural basis for the unique and well‐documented functional property of the P2X7 receptor, namely, lack of receptor desensitization even following prolonged activation.^11^ The distal C‐terminus of each subunit, including β_16_–β_18_ and α_9_–α_16_, forms a cytoplasmic ballast (Figure 1A) that hangs beneath the transmembrane part of an adjacent subunit. Intriguingly, the structures of the rat P2X7 receptor disclosed one GDP‐binding site and two Zn^2+^‐binding sites in each of the cytoplasmic ballasts. The implications of structural features to receptor function remain elusive (Figure 1A), even though the P2X7 receptor is known for its sensitivity to inhibition by Zn^2+^ extracellularly.^71^, ^72^ Overall, the structural domains revealed by the structures of the rat P2X7 receptor are highly conserved in mammalian P2X7 receptors (see our recent review^73^ for more details).

The ATP‐bound structure of the rat P2X7 receptor, together with the ATP‐bound structures of the zebrafish P2X4 and human P2X3 receptors, has consistently revealed three ATP‐binding sites located at the three intersubunit interfaces (Figure 1B,C), as proposed by earlier biochemical and functional studies.^14^ Moreover, these structures have delineated the ATP‐binding pocket, particularly the conserved hydrophilic residues coordinating ATP binding site at the apex, which were identified by site‐directed mutagenesis studies.^14^, ^15^ In the rat P2X7 receptor, Lys^64^, Lys^66^, and Thr^189^ in the lower body from one subunit and Asn^292^, Arg^294^, and Lys^311^ in the upper body of the neighboring subunit participate in ATP binding (Figure 1C), and these residues are conserved in mammalian P2X7 receptors.^73^ Detailed examination of the structures of the zebrafish P2X4, human P2X3 and rat P2X7 receptors in the apo, closed and ATP‐bound, open states infer the conformational changes that accompany receptor activation or channel gating.^14^, ^65^, ^67^ Occupation by ATP of its binding site drives outward movement of the lower body and opening of the three lateral fenestrations located above the plasma membrane as the ion entrance or exit portals (Figure 1B). The resulting energy is transduced to the transmembrane part via the structure termed the connecting rods formed by multiple β‐sheets in the upper body and lower body^14^ and induces relative movement of the TM1 and TM2 domains and outward flexing of the TM2 domains, opening the ion‐permeating pathway (Figure 1B).

The P2X7 receptor, as introduced above, has an eminent ability to induce large pore formation. Experimentally, this functionality was shown by the shift in equilibrium potential which was interpreted to indicate an increase in the permeability to organic cations like N‐methyl‐d‐glucamine (NMDG^+^). However, this methodology has been called into question, because, as shown for the P2X2 receptor, such apparent shift should be attributed to substantial increase in intracellular concentration of NMDG^+^ that flows into the cell during prolonged receptor activation, rather than a change in the permeability to NMDG^+^ of the open channel.^74^ A more reliable but mechanistically less informative method to monitor large pore formation is to image uptake of extracellular membrane‐impermeable, DNA‐binding fluorescent dyes, such as YO^−^PRO‐1^2+^ or ethidium^+^.^19^ Accumulating evidence supports the importance of large pore formation for the role of the P2X7 receptor, for example, in chronic pain, multiple sclerosis, macular degeneration, and osteoporosis,^75^, ^76^, ^77^, ^78^ as well as microglial production of interleukin (IL)‐1β.^79^ However, the pore‐forming mechanism remains the most elusive aspect of the P2X7 receptor. Two hypotheses among others have attracted considerable attention.^11^, ^80^, ^81^ The first one is that the pannexin‐1 channel couples to the P2X7 receptor and prolonged P2X7 receptor activation induces the pannein‐1 channel to open and form the large pore. The second one is that the P2X7 receptor ion channel, upon sustained activation, undergoes a progressive increase in its size.^82^ However, such a dilatation mechanism has been challenged.^83^ Nonetheless, prolonged P2X7 receptor activation can induce an NMDG^+^‐permeable open state that functionally differs from the open state, only permeating small cations.^84^ In endorsing the notion that the P2X7 receptor can form a large pore, a recent study showed that the purified giant panda P2X7 receptor, after reconstitution into liposomes, formed a large pore in a manner depending on the membrane lipid composition.^85^ Like the P2X7 receptor, the TRPV1 channel displays an increase in the apparent permeability to large organic cations upon extended activation.^86^, ^87^ A recent study reveals that the TRPV1 channel, with dynamic changes in the outer pore and ion selectivity filter, can adopt a distinctive conformation that enables the permeation of NMDG^+^.^88^ This finding is remarkable, providing the first structural evidence to indicate that an ion channel can have a tuneable ion permeability. Future structural studies may help clarify whether the P2X7 receptor ion channel can possesses an adaptive ion permeability and form the large pore.

In short, the structures of the P2X7 and other P2X receptors in the apo, closed state and ATP‐bound, open state, reveal ATP binding and inform of channel gating principle, consistent with earlier biochemical, functional and mutational studies. The structures also begin to provide additional insights into subtype‐specific functional properties. We anticipate that studies combining structural determination and modeling, mutagenesis and functional characterization will continue to evolve our understanding of the modus operandi of the P2X receptors, including P2X7 receptor‐dependent large pore formation.

## 3 P2X7 RECEPTOR IN TRAUMATIC CNS DAMAGE CONDITIONS AND RELATED COMPLICATIONS

### 3.1 SCI

Traumatic SCI is a highly destructive CNS damage condition, caused mostly by motor vehicle accidents and falls. The pathophysiology of SCI is complex, with the initial physical damage to the spinal cord eliciting a cascade of pathogenic events, including ATP release and tissue inflammation, that interdependently cause secondary injury that affects functional recovery.^89^ Several studies examined the ATP‐P2X7 receptor signaling axis in secondary injury following SCI (Table 1). In rats with contusive SCI induced by weight‐drop, apoptotic cell death happened to neurons and oligodendrocytes in the area surrounding the lesion site, particularly in the regions with a high level of ATP release.^90^ In supporting such cytolytic effects arising from ATP‐induced P2X7 receptor activation, application of ATP, ATPγS or 2ʹ(3ʹ)‐*O*‐(4‐benzoylbenzoyl)‐ATP (BzATP), which all can activate the P2X7 receptor, induced apoptotic cell death in healthy spinal cord. In contrast, the application of 2‐methylthio‐ATP (2MeSATP) or α,β‐methyleneadenosine ATP (αβmeATP), which are ineffective at the P2X7 receptor, resulted in minimal cell death. SCI‐induced apoptotic cell death was reduced and motor function recovery improved by treatment before SCI with oxidized ATP (oxATP), which has multiple actions including irreversible inhibition of the P2X7 receptor,^91^ or treatment shortly after SCI with pyridoxal‐5ʹ‐phosphate‐6‐azophenyl‐2ʹ,4ʹ‐disulphonic acid (PPADS), a generic P2 antagonist.^90^ As shown in a follow‐up study,^92^ SCI‐induced tissue damage was attenuated and motor function recovered better in rats by systemic administration of Brilliant Blue G (BBG), a P2X7 receptor‐specific antagonist, given daily for 3 consecutive days after SCI. Similarly, tissue damage was mitigated and motor function preserved by intraperitoneal injection of BBG every 12 h for 3 days, starting shortly after SCI in rats subjected to SCI induced by compression.^93^ Thus, studies using different rodent models provide consistent evidence to support that the ATP‐P2X7 receptor signaling axis plays a critical role in mediating secondary injury induced by SCI and suggest that inhibition of the P2X7 receptor facilitates functional recovery (Table 1).

**Figure 2:**
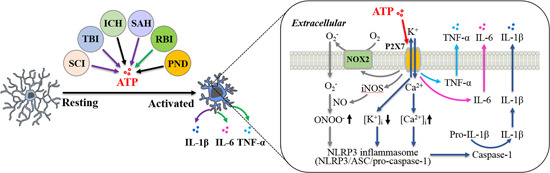
P2X7 receptor in microglia mediates neuroinflammation after traumatic CNS damage. Various traumatic CNS damage conditions, including spinal cord injury (SCI), traumatic brain injury (TBI), intracerebral hemorrhage (ICH), subarachnoid hemorrhage (SAH), radiation‐induced brain injury (RBI), and perioperative neurocognitive dysfunction (PND), causes ATP release from injured tissues that induces microglial activation and generation of proinflammatory cytokines, interleukin (IL)‐1β, IL‐6 and tumor necrosis factor (TNF)‐α, leading to neuroinflammation. In the inset: Summary of the molecular mechanisms for ATP‐induced P2X7 receptor‐mediated generation of IL‐1β, IL‐6, and TNF‐α by microglia following traumatic CNS damage conditions. ATP‐induced P2X7 receptor activation induces K^+^ efflux or Ca^2+^ influx through the P2X7 receptor ion channel and thereby activates the NLRP3 inflammasome. Alternatively, P2X7 receptor activation stimulates the expression and activity of NADPH oxidase 2 (NOX2) to generate O_2_^‐^ and inducible NO synthase (iNOS) to generate NO, leading to formation of peroxynitrite (ONOO^‐^) that in turns activates the NLRP3 inflammasome. Activation of the NLRP3 inflammasome activates caspase‐1, which converts proIL‐1β to mature IL‐1β. ATP‐induced P2X7 receptor activation also stimulates generation of IL‐6 and TNF‐α. ATP, adenosine 5ʹ‐triphosphate; CNS, central nervous system. [Color figure can be viewed at wileyonlinelibrary.com]

There is compelling evidence that microglia‐mediated neuroinflammation strongly influences the development of secondary injury after SCI.^94^, ^95^ Activation of the NOD‐, leucine‐rich repeat‐, and pyrin domain‐containing protein 3 (NLRP3) inflammasome represents one major mechanism activating caspase‐1 to produce IL‐1β, the key proinflammatory cytokine in the initiation of innate immune response. The NLRP3 inflammasome is a multiprotein complex composed of the NLRP3, adapter protein apoptosis‐associated speck‐like protein containing a CARD (caspase activation and recruitment domain) (ASC), and procaspase‐1.^96^ The P2X7 receptor has been well established for its key role in ATP‐induced activation of the NLRP3 inflammasome and caspase‐1 in immunocompetent cells,^97^, ^98^ via mediating Ca^2+^ influx to elevate intracellular Ca^2+^ concentration or, as has been recently proposed, K^+^ efflux to reduce intracellular K^+^ concentration.^99^, ^100^, ^101^ Microglial activation occurred in rats after contusive SCI, which was suppressed by systemic and repetitive administration of BBG.^92^ The expression of NLRP3, ASC, and caspase‐1, as well as P2X7 receptor, in the rat spinal cord was upregulated after SCI, which was inhibited by intraperitoneal injection of BBG after SCI.^93^ Recent studies provide evidence to show upregulation of microglial P2X7 receptor expression in spinal cord in rats following SCI induced by bilateral dorsal lesion^102^ or contusive SCI.^103^ Moreover, such upregulation of microglial P2X7 receptor expression was mitigated by intraperitoneal application of BBG or A‐438079, another P2X7 receptor‐specific antagonist, and, conversely, enhanced by intraperitoneal application of BzATP.^102^, ^103^ Importantly, SCI‐induced upregulation of the NLRP3 expression, activation of caspase‐1, and production of IL‐1β were inhibited by treatment with A‐438079.^102^ Collectively, these findings support an important role for microglial P2X7 receptor in the spinal cord in mediating neuroinflammation after SCI via activation of the NLRP3 inflammasome and caspase‐1 and production of IL‐1β (Figure 2).

Studies also show that the P2X7 receptor plays a role in mediating other pathogenic events following SCI. Reactive astrogliosis, characteristic of hypertrophic astrocytes with multiple processes expressing glial fibrillary acidic protein (GFAP), and neutrophil infiltration into the peri‐lesion area were observed in rats after contusive SCI, all of which were suppressed by systemic administration of BBG.^92^ Blood‐spinal cord barrier function was impaired in rats after contusive SCI, which was attenuated by intraperitoneal injection of BBG.^93^ However, the cellular and molecular mechanisms by which the P2X7 receptor mediate these pathogenic events remain unclear. It is perhaps worth mentioning the notion proposed by some studies that activation of the P2X7 receptor in spinal cord neurons mediates neuronal cell death but neuronal P2X7 receptor expression in the CNS, as mentioned above, remains debatable.

SCI or, more specifically, SCI‐induced neuroinflammation can cause detrimental effects on other tissues and organs, leading to the development of chronic complications in people living with SCI. There is a high rate of prevalence of chronic pain, with neuropathic pain reported as the most common and distressing condition.^104^, ^105^, ^106^ Upregulation of microglial P2X7 receptor expression in the spinal cord is known to amplify or exacerbate neuropathic and inflammatory pains due to damage and inflammation of peripheral tissues.^107^, ^108^, ^109^, ^110^, ^111^ Further studies are required to dissect whether the upregulated expression and activity of microglial P2X7 receptor in the spinal cord contribute to SCI‐induced chronic pain. It is interesting to notice that SCI induced by transient ischemia and reperfusion (I/R), a common complication of spine, thoracic and cardiovascular operations, is accompanied with a high incidence of pain hypersensitivity. The development of heightened pain, shown in a recent study after I/R‐induced SCI using transient aortic arch occlusion in mice, strongly correlates with the increase in P2X7 receptor expression in the spinal cord.^112^ I/R‐induced SCI resulted in activation of caspase‐1 and the production of IL‐1β in the spinal cord. The increase in P2X7 receptor expression, activation of caspase‐1, and production of IL‐1β were prevented by intrathecal injection of A‐438079, and the pain hypersensitivity suppressed by treatment with A‐438079 or an anti‐IL‐1β antibody before I/R‐induced SCI.^112^ Interestingly, as revealed in the same study, the expression of microRNA (miR)‐187‐3p in the spinal cord was downregulated after I/R‐induced SCI. Intrathecal injection of a miR‐187‐3p mimic that reduced the P2X7 receptor expression also alleviated I/R‐induced activation of caspase‐1, production of IL‐1β, and pain sensitivity. On the contrary, intrathecal injection of a miR‐187‐3p inhibitor that enhanced the P2X7 receptor expression also stimulated I/R‐induced activation of caspase‐1, production of IL‐1β, and pain sensitivity. Thus, the P2X7 receptor in spinal cord plays a vital role in mediating neuroinflammation in the development of pain hypersensitivity following I/R‐induced SCI.^112^ There is a greater prevalence of neurodegenerative and neurological conditions in SCI patients, with suppression by inflammation of hippocampal neurogenesis being proposed as a common factor.^113^ It remains unclear whether SCI‐induced P2X7 receptor‐mediated neuroinflammation contributes to the inhibition of hippocampal neurogenesis. Neurogenic bladder dysfunction is another debilitating complication of SCI. The increased expression and activity of the urothelial P2X3 receptor plays a critical role in SCI‐induced overactive bladder.^114^ SCI‐induced upregulated P2X3 receptor expression in rats was prevented and bladder dysfunction improved by localized application of BBG at the injury site to inhibit SCI‐induced microglial activation in the spinal cord.^102^

In summary, studies provide evidence to support an important role for the P2X7 receptor in mediating SCI‐induced secondary injury and chronic complications, such as chronic pain and neurogenic bladder dysfunction (Table 1). Specifically, the P2X7 receptor in microglia mediates neuroinflammation in SCI‐induced second injury and the development of chronic complications via activation of the NLRP3 inflammasome and caspase‐1 to produce IL‐1β (Figure 2).

### 3.2 TBI

TBI, mainly resulting from violent forces on the head, is a leading cause of death and disability for people under the age of 45 years, with a high incidence in young men. Like traumatic SCI, TBI evolves secondary injury with complicated pathophysiology.^115^ There is compelling evidence that microglia play a key part in TBI‐induced secondary injury and, more specifically, chronic microglial activation and microglia‐mediated neuroinflammation drive neurodegeneration and impair motor and cognitive functions following severe TBI.^116^, ^117^, ^118^, ^119^, ^120^, ^121^

As revealed in an earlier study using two‐photon imaging of microglia in intact mouse cortex and after laser ablation‐induced or mechanically induced TBI, the fine processes of microglia exhibited high dynamics in healthy brain and were rapidly mobilized into the injury site to form a barrier around the injured tissue.^122^ Microglia displayed similar responses to local application of ATP in healthy brain. TBI‐induced chemotactic response of microglia was lost in the presence of apyrase, an ATP‐scavenging enzyme, and PPADS. Such chemotactic response of microglia was also eliminated by the application of flufenamic acid (FFA), which preferentially inhibits the connexin channels known to be expressed in astrocytes and mediate ATP‐induced ATP release from astrocytes.^122^ As shown in a more recent study, tissue damage, and motor function deficits in mice after controlled cortical impact (CCI)‐induced TBI were mitigated by intraperitoneal injection of trovafloxacin, a broad‐spectrum antibiotic that can block the pannexin‐1 channel.^123^ TBI‐induced loss of blood‐brain barrier (BBB) function was reduced by treatment with trovafloxacin. Moreover, the levels of IL‐1β, IL‐6, and tumor necrosis factor (TNF)‐α, the major proinflammatory cytokines, were lowered upon treatment with trovafloxacin, which well correlated with reduced immune cells infiltrated in the injury area. As demonstrated in vitro using murine BV2 microglial cells, both ATP release and cell migration induced by chemotactic stimulation were prohibited by treatment with trovafloxacin.^123^ Taken together, these studies suggest that TBI causes ATP release from microglia through the pannexin‐1 channel and further ATP‐induced ATP release from astrocytes through the connexin channel and that ATP induces microglial activation, BBB dysfunction, and infiltration of peripheral immune cells.

Several studies have pointed to the P2X7 receptor for its important role in mediating neuroinflammation after CCI‐induced TBI.^124^, ^125^, ^126^ Microglial P2X7 receptor expression was upregulated in the rat cerebral cortex after TBI.^125^, ^126^ TBI‐induced IL‐1β expression and neurological dysfunction were attenuated in mice by intravenous injection of BBG via tail vein after TBI, or by genetic depletion of the P2X7 receptor expression.^124^ TBI‐induced IL‐1β expression in the cortex in rats was also reduced and neurological function improved by intravenous treatment with BBG after TBI.^125^ As shown in another recent study in rats, microglial activation and generation of IL‐1β in the cerebral cortex were reduced and neurological function improved by intraperitoneal injection of A‐804598 immediately after TBI.^126^ These findings are consistent with activation of the NLRP3 inflammasome in microglia after CCI‐induced TBI,^117^ together supporting the notion that activation of the P2X7 receptor in microglia induces production of IL‐1β via activation of the NLRP3 inflammasome and caspase‐1, but further studies are required to support this mechanism. There is evidence to show that CCI‐induced TBI caused microglial shedding of microvesicle‐like particles and apoptotic neuronal cell death in the injured and adjacent regions of cerebral cortex in rats, and such effects were largely blocked by intraperitoneal injection of A‐804598 after TBI.^126^ Interestingly, as reported by another recent study in rats after CCI‐induced TBI, activated microglia released microparticles that enrich with proinflammatory mediators, and seeding such microglia‐derived microparticles in healthy rat brains induced microglial activation and production of IL‐1β and TNF‐α.^127^ It remains however unclear whether the P2X7 receptor plays a role in mediating microglial release of such proinflammatory microparticles.

There is also evidence to suggest that the P2X7 receptor has a role in mediating reactive astrogliosis and edema resulting from CCI‐induced TBI. Reactive astrogliosis in the cerebral cortex in rats was suppressed by intraperitoneal injection of A‐804598 after TBI.^126^ Cerebral edema contributes to elevation of the intracranial pressure and thus represents a common life‐threatening complication. TBI‐induced cerebral edema formation was inhibited in mice by intravenous administration of BBG after TBI and lessened in P2X7 receptor‐deficient mice.^124^ Similarly, cerebral edema in rats was subdued by intravenous injection of BBG after TBI.^125^ Specifically, TBI‐induced expression of GFAP, and aquaporin‐4, a water channel in astrocytes that is known to mediate edema formation, was reduced by treatment with BBG, leading to the suggestion that the P2X7 receptor in astrocytes plays a major role in mediating edema formation following TBI.^124^

In summary, increasing evidence supports that ATP‐induced signaling plays an important role in secondary injury after TBI. The P2X7 receptor in microglia mediates neuroinflammation via inducing production of IL‐1β (Figure 2) and microvesicle‐like particles, and the P2X7 receptor in astrocytes is engaged in edema formation via upregulating the expression of aquaporin‐4 water channel (Table 1). More studies are required for better understanding P2X7 receptor‐mediated mechanisms in TBI.

### 3.3 IS

The brain has a high demand for energy and thus is metabolically active, making it easily prone to structural and functional damage by limiting oxygen and glucose supply. Stroke is the major cause of severe and chronic disability, as well as a leading cause of death, in adults, and 80%–85% of stroke cases are IS, resulting from blockage of a blood vessel in the brain.^128^, ^129^ Loss of blood flow to the brain, for example, due to cardiac arrest, can also cause brain ischemia. Available emergency procedures with medications mainly aim to restore blood circulation to prevent brain damage and fatal consequences by severe or prolonged ischemia. Reperfusion following transient ischemia can also incur brain damage, which is often termed I/R‐induced or postischemia brain damage that contributes to loss of neurological and cognitive functions in stroke patients.^129^

There is clear evidence to support an important role for the ATP‐P2X7 receptor signaling axis in brain damage during ischemia, particularly cell death triggered by ATP‐induced P2X7 receptor‐mediated disruption of intracellular Ca^2+^ homeostasis (Table 1). Exposure of mouse neonatal hippocampal slices to oxygen and glucose deprivation (OGD), an in vitro model of global ischemia, caused microglial cell death, which was reduced by treatment with apyrase or BBG, or by removal of extracellular Ca^2+^ to prevent an increase in intracellular Ca^2+^ concentration.^130^ Consistently, there was significantly reduced OGD‐induced microglial cell death in neonatal hippocampal slices from P2X7 receptor‐deficient mice.^130^ Exposure to OGD also induced cell death in rat oligodendrocytes in culture or oligodendrocytes in optic nerve preparations from mice and rats and, OGD‐induced oligodendrocyte death was suppressed by treatment with PPADS or BBG, or by removal of extracellular Ca^2+^.^131^ In addition to cell death, exposure to OGD induced oligodendrocytes to release ATP. OGD‐induced ATP release and cell death were inhibited by treatment with mefloquine (MFQ), a pannexin channel inhibitor, FFA or carbenoxolone (CBX), a connexin channel blocker.^131^ Furthermore, exposure of optic nerve preparations to OGD caused severe myelin damage, which was attenuated by treatment with MFQ, FFA, CBX, PPADS or BBG and, loss of white matter function, which was significantly recovered by treatment with PPADS or BBG, or by removal of extracellular Ca^2+^.^131^ Therefore, ischemia induces release of ATP from oligodendrocytes, which acts as an autocrine or paracrine signal to activate the P2X7 receptor to disrupt intracellular Ca^2+^ homeostasis, leading to cell death, myelin damage and impairment in white matter function. Moreover, exposure to OGD elicited an irreversible loss of excitatory synaptic transmission in granule neurons in rat hippocampal slices, which was prevented by treatment with BBG.^132^ Such results support engagement of the P2X7 receptor in OGD‐induced ischemia brain damage via disruption of synaptic function but the underlying mechanism is less clear.

Evidence also exists to support that the ATP‐P2X7 receptor signaling axis plays a significant role in mediating I/R‐induced or postischemia brain damage (Table 1). Infarction, neuronal loss in the cerebral cortex and neurological dysfunction in rats, due to reperfusion after transient ischemia induced by middle cerebral artery occlusion (MCAO), an in vivo model of IS, were mitigated by intraperitoneal administration of BBG twice a day, starting shortly after ischemia.^133^ Similarly, infarction, neuronal cell death and neurological deficits in mice due to reperfusion after transient MCAO were reduced by intraperitoneal injection of MFQ daily or BBG twice a day, beginning shortly after ischemia.^134^ Of note, there was no additive effect in reducing postischemia brain damage by combined treatment with MFQ and BBG, supporting that brain damage is mediated by the same signalling mechanism, namely, the ATP‐P2X7 receptor signaling axis.^134^ Consistently, infarction, neuronal cell death and neurological deficits were significantly less in pannexin‐1‐deficent mice or P2X7 receptor‐deficient mice.^134^ Under in vitro conditions, neuronal cell death in rat brain slices, 24 h after exposure to transient OGD, was reduced by treatment with BBG.^133^ Similarly, there was less neuronal cell death in mouse cortical slices, also examined 24 h after transient OGD, by treatment with MFQ or BBG, and in slices from pannexin‐1‐deficent mice or P2X7 receptor‐deficient mice.^134^ Collectively, these studies provide strong evidence to demonstrate the importance of the ATP‐P2X7 receptor signaling axis in mediating postischemia brain damage.

It remains less clear regarding the role of the P2X7 receptor in microglia‐mediated neuroinflammation in either ischemia‐induced or postischemia brain damage. Nonetheless, the number of microglia in the cortex was increased, and such cell proliferation occurred earlier than microglial cell death in rat brain slices during reperfusion after transient OGD.^135^ Interestingly, postischemia microglial cell death was prevented by treatment with indomethacin, an anti‐inflammatory agent inhibiting cyclooxygenase, or treatment with PPADS and, conversely, worsened in brain slices cocultured with murine N9 microglia. Prolonged exposure of rat brain slices to a high concentration of ATP induced massive cell death in the cortex and striatum.^135^ Exposure of rat brain slices to OGD‐induced transient ischemia and perfusion resulted in an increase in microglial P2X7 receptor expression in the cortex. However, the role of the P2X7 receptor in microglia remains inadequately understood. It is worth mentioning the suggestion from earlier studies that the P2X7 receptor in neurons mediates neuronal cell death after transient OGD, but the neuronal P2X7 receptor expression remains controversial.

In summary, there is evidence to indicate that the ATP‐P2X7 receptor signaling axis plays an important role in both brain damage during ischemia and brain damage during reperfusion after transient ischemia (Table 1). In particular, prolonged ischemia induces release of ATP, which activates the P2X7 receptor and disrupts intracellular Ca^2+^ homeostasis in microglia and oligodendrocytes to cause their demise. Given the unpredictable nature of IS, postischemia brain damage is more amenable to intervention. Thus, it is interesting to explore the P2X7 receptor as a potential target for mitigating postischemia neurological and cognitive dysfunction in IS patients. More investigations are required for better understanding the role of the P2X7 receptor in microglia in mediating neuroinflammation in IS brain damage.

### 3.4 Hemorrhagic stroke

Hemorrhagic stroke contributes 15%–20% of stroke cases and has two main types; intracranial hemorrhage (ICH), also known as intracerebral hemorrhage due to burst of a cerebral blood vessel, and subarachnoid hemorrhage (SAH) mainly caused by rupture of a blood vessel with blood leaking into the subarachnoid space.^128^, ^136^, ^137^

Recent studies have gathered substantial evidence to indicate an important role for the P2X7 receptor in ICH brain damage in rats and mice induced by injecting collagenase^138^, ^139^, ^140^, ^141^, ^142^ or autologous blood^140^, ^143^ (Table 1). In these studies, ICH‐induced apoptotic neuronal cell death and neurological deficits were attenuated by intracerebroventricular injection of P2X7 receptor‐specific siRNA before ICH, or intraperitoneal administration with BBG or A‐438079 after ICH.^138^, ^140^, ^142^, ^143^ Neuroinflammation mediated by activation of the NLRP3 inflammasome and production of IL‐1β is an important mechanism in ICH brain damage.^144^ Consistently, there is evidence to support critical involvement of the P2X7 receptor in mediating neuroinflammation following ICH. As shown in rats, ICH led to upregulation of P2X7 receptor expression, microglial activation, expression of IL‐1β, IL‐6, and TNF‐α, upregulation of GFAP expression and neuronal cell death, which were mitigated by perfusion of BBG, 6 h after ICH.^139^ Similarly, the expression of P2X7 receptor, NLRP3 and ASC in microglia was upregulated, and the NLRP3 inflammasome became activated in rats after ICH. Such ICH‐induced upregulation of the NLRP3 and ASC expression, activation of the NLRP3 inflammasome and production of IL‐1β were ameliorated by intracerebroventricular injection of P2X7‐siRNA before ICH, or by intraperitoneal injection of BBG 1 day or even 3 days after ICH.^138^ Mechanistically, activation of the P2X7 receptor stimulates the expression and activity of NADPH oxidase 2 (NOX2) and inducible nitric oxide synthase (iNOS) in microglia, leading to generation of peroxynitrite, which in turn activates the NLRP3 inflammasome and caspase‐1 (Figure 2).

Hydrogen sulfide (H_2_S) is a gaseous signaling molecule known for its neuroprotective role in the CNS via multiple actions, including anti‐inflammation.^145^ The brain H_2_S level and microglial expression of cystathionine‐β‐synthase (CBS), the major H_2_S‐producing enzyme in the brain, were reduced in rats after ICH.^141^ ICH‐induced microglial activation, apoptotic neuronal cell death, edema formation and neurological deficits were consistently attenuated by treatment with S‐adenosyl‐l‐methionine (SAM), a CBS agonist, or sodium hydrosulphide (NaHS), one of the commonly used H_2_S donors, via downregulating microglial P2X7 receptor expression.^141^ Exposure of cultured rat microglia to LPS, which is the major outer surface membrane component present in almost all Gram‐negative bacteria and is a PAMP widely used in in vitro and in vivo studies, and ATP led to upregulation of P2X7 receptor, NLRP3 and ASC expression, activation of the NLRP3 inflammasome and production of IL‐1β, and all these effects were inhibited by treatment with SAM and NaHS.^141^ Collectively, these results support a novel mechanism for H_2_S‐induced neuroprotection and also show a vital role of the P2X7 receptor in microglia in mediating activation of the NLRP3 inflammasome and production of IL‐1β in ICH brain damage.

Studies also support a role for the P2X7 receptor in mediating ICH‐induced impairment in BBB function and neutrophil infiltration (Table 1). ICH‐induced neutrophil infiltration in rats was mitigated by treatment with BBG.^138^, ^141^ Interestingly, in rats with autologous blood injection‐induced ICH, the expression of occudin, vascular endothelial (VE)‐cadherin and zonula occluden‐1 (ZO‐1), the major proteins forming endothelial junction, was downregulated, and the BBB function impaired. These effects were reduced by treatment with P2X7‐siRNA or A‐438079.^140^ Furthermore, the guanosine‐5'‐triphosphate (GTP)‐RhoA activity was enhanced, which was also reduced by treatment with P2X7‐siRNA or BBG. ICH‐induced reduction in the expression of endothelial junction proteins, BBB dysfunction and neurological deficits were ameliorated by treatment with C3 transferase, a RhoA inhibitor.^140^ Similarly, the expression of occudin was reduced and the BBB function impaired in rats after collagenase injection‐induced ICH, which were mitigated by treatment with A‐438079.^140^ Thus, the P2X7 receptor in endothelial cells mediates ICH‐induced RhoA activation and subsequent downregulation of the endothelial junction protein expression, leading to compromised BBB integrity and brain damage.^140^

Recent studies have revealed engagement of mitogen‐activated protein kinases (MAPK), p38, extracellular signal‐regulated kinase (ERK) and Jun N‐terminal kinase (JNK) and, furthermore, activation of caspase‐3 and nuclear factor kappa B (NF‐κB) in neurons in rats after autologous blood injection‐induced ICH^143^ and in mice after collagenase injection‐induced ICH.^142^ In rats with autologous blood injection‐induced ICH, such effects were enhanced by treatment with BzATP and, conversely, were prevented by treatment with P2X7‐siRNA. BzATP‐induced activation of p38 and caspase‐3 was suppressed by treatment with a p38 inhibitor, whereas BzATP‐induced activation of ERK and NF‐kB was reduced by treatment with nimbolide, an ERK inhibitor. These findings led to the suggestion that activation of the P2X7 receptor in neurons induces activation of the p38 and caspase‐3, a key signaling molecule in caspase‐dependent apoptosis, and ERK/NF‐κB signaling pathways to trigger apoptotic neuronal cell death following ICH.^143^ Following collagenase injection‐induced ICH in mice,^142^ upregulation of the P2X7 receptor expression, apoptotic cell death, edema and neurological deficits were attenuated by intraperitoneal injection of A‐438079 and, conversely, were exacerbated by intraventricular injection of BzATP. ICH‐induced ERK/NF‐kB activation was attenuated by treatment with A‐438079 and, by contrast, was enhanced by treatment with BzATP. Moreover, the NOX2 expression was upregulated following ICH leading to increased ROS production, which was reduced by treatment with A‐438079. Therefore, it was proposed that activation of the P2X7 receptor in neurons induces oxidative stress to cause neuronal cell death and brain damage after ICH, via stimulating the ERK/NF‐κB signaling pathway to upregulate neuronal NOX2 expression.^142^ However, the P2X7 receptor expression in CNS neurons remains uncertain.

The thalamocingulate circuitry is a medial pathway of pain processing, and thus hemorrhagic stroke, if occurring to the lateral thalamic region, can induce central poststroke pain (CPSP). As shown in a recent study examining the role of the P2X7 receptor in CPSP in rats after collagenase injection‐induced ICH,^145^ the aberrant or excessive neuronal activity of anterior cingulate cortex and medial thalamus in response to sciatic nerve stimulation was suppressed, and the locomotor activity restored by treatment with BBG or A‐740003. CPSP was alleviated by treatment with BBG or an anti‐IL‐1β antibody. Mechanistically, P2X7 receptor‐mediated microglial production of IL‐1β stimulates release of glutamate to cause hyperexcitability of neurons in medial thalamus and anterior cingulate cortex in response to nociceptive inputs, leading to the development of CPSP.^145^

There is evidence to suggest that the P2X7 receptor plays a critical role in mediating brain damage by SAH (Table 1). Apoptotic neuronal cell death, edema formation and neurological dysfunction in rats after endovascular perforation‐induced SAH were ameliorated by intracerebroventricular administration of P2X7‐siRNA before SAH, or intraperitoneal injection of BBG shortly after SAH.^146^, ^147^ Such genetic or pharmacological inhibition of the P2X7 receptor attenuated activation of p38 and caspase‐3.^146^ SAH‐induced neutrophil filtration was decreased by treatment with BBG.^147^ In addition, SAH‐induced activation of caspase‐1 and production of IL‐1β were reduced by treatment with P2X7‐siRNA or BBG, as well as treatment with NLRP3‐siRNA.^147^ Thus, the P2X7 receptor mediates neuroinflammation, via activation of the NLRP3 inflammasome and caspase‐1 and production of IL‐1β (Figure 2) and other pathogenic events to drive brain damage after SAH.

### 3.5 NHI

NHI is the common factor causing death and disability in human neonates and is associated with the development of motor and cognitive dysfunction in later life.^148^ Hypoxic‐ischemic encephalopathy (HIE) is an NHI‐related condition, manifesting neonatal seizures. Clinical findings and preclinical studies suggest neonatal seizures can lead to brain injury. As reported in a recent study,^149^ the overall P2X7 receptor expression was several times higher in neocortex samples from 3 months old patients with HIE and seizures, as compared with age‐matched healthy subjects. The P2X7 receptor expression in the neocortex and hippocampus, 24 h after exposure of 7‐day‐old mouse pups to transient global hypoxia, was also upregulated.^149^ Hypoxia‐induced neonatal seizures in mouse pups were suppressed by intraperitoneal injection of A‐438079 or JNJ‐47965567, another P2X7 receptor‐specific antagonist, before exposure to hypoxia. Exposure to hypoxia also induced microglial activation, increased IL‐1β mRNA expression and procapase‐1 protein expression, and activation of caspase‐1. These effects were prevented by treatment with A‐438079.^149^ Therefore, the P2X7 receptor in microglia plays a role in neuroinflammation via mediating activation of capase‐1 in response to hypoxia (Table 1). Clearly, investigations are required to examine the role of the P2X7 receptor in microglia in mediating activation of the NLRP3 inflammasome and production of IL‐1β, and, furthermore, the contribution of P2X7 receptor‐mediated neuroinflammation in brain damage due to NHI and related HIE.

### 3.6 RBI

RBI occurs in patients receiving radiotherapy treatment for head and neck cancers, and results from radiation‐induced damage of the adjacent healthy brain tissues that leads to cognitive complications.^150^, ^151^ As reported in a recent study, the ATP level in cerebrospinal fluid (CSF) from RBI patients was several times higher than that in CSF from patients without RBI.^152^ The levels of proinflammatory mediators, including cyclooxygenase‐2 (COX‐2), IL‐6, and TNF‐α, were also elevated in CSF from RBI patients. Importantly, there were strong positive correlations of the CSF ATP level with the CSF level of COX‐2, IL‐6, and TNF‐α, particularly with the severity of RBI. Consistently, the ATP level in CSF was higher 3 days in mice with whole brain irradiation and continued to rise 7 days postirradiation. Irradiated mice exhibited loss of neurons in the cortex and impaired neuronal proliferation in the dentate gyrus region, examined 14 days after irradiation, and cognitive dysfunction, examined 8 weeks after irradiation. Irradiation‐induced loss of cortical neurons, impairment in neurogenesis, and cognitive dysfunction were attenuated by intraperitoneal injection of BBG for consecutive 7 days, starting 2 h before irradiation, supporting an important role for the P2X7 receptor in mediating RBI.^152^ Consistent with the finding in RBI patients that neuroinflammation is critical in RBI pathogenesis is that microglia were activated and the expression of COX‐2, IL‐6, and TNF‐α, as well as P2X7 receptor in microglia, was increased in irradiated mice. Microglial activation and increased expression of P2X7 receptor, COX‐2, IL‐6, and TNF‐α were attenuated in mice treated with BBG. Under in vitro conditions, exposure to radiation of cultured mouse microglia, astrocytes and neurons caused ATP release. Exposure of cultured microglia to radiation also resulted in microglial activation, production of COX‐2, IL‐6, and TNF‐α, and upregulated P2X7 receptor expression, and induced apoptosis of hippocampal neurons cocultured with microglia. These effects were inhibited by treatment with BBG or P2X7‐siRNA before radiation and, conversely, were enhanced by application of ATP. Taken together, these results support an important role for the ATP‐P2X7 receptor signaling axis in microglia in mediating neuroinflammation via production of proinflammatory mediators (Figure 2).

### 3.7 PND

PND, an umbrella term embracing a spectrum of postoperative cognitive dysfunction after anesthesia and surgery, is the most common but least recognized complication in older patients. Microglial activation and neuroinflammation have been identified as critical pathogenic events in the development of PND.^153^, ^154^, ^155^ A recent study has examined the role of the P2X7 receptor in PND pathogenesis, particularly microglia‐mediated neuroinflammation, in a mouse model induced by right common carotid arterial surgery under anesthesia.^156^ Anesthesia and surgery upregulated microglial P2X7 receptor expression and caused cognitive dysfunction, examined 2 weeks after surgery. Such cognitive dysfunction was prevented by intraperitoneal injection of BBG for 7 days, starting just before surgery, and was absent in P2X7 receptor‐deficient mice (Table 1). Anesthesia and surgery also induced microglial activation, upregulation of the precaspase‐1 expression, activation of caspase‐1, and increased IL‐1β level in the hippocampus. These effects were reduced by treatment with BBG or by deficiency in P2X7 receptor expression, strongly supporting an important role for the P2X7 receptor in microglia in the pathogenesis of PND via neuroinflammation by activation of capase‐1 and production of IL‐1β (Figure 2). In agreement with activation of the NLRP3 inflammasome in mediating P2X7 receptor‐dependent activation of caspase‐1, a recent study using aged mice (18 months old) shows that neurological dysfunction due to anesthesia and surgery was reduced by intraperitoneal injection of MCC950, an NLRP3 inflammasome inhibitor, just before surgery and daily for the following 2 days.^157^ In addition, activation of microglia and astrocytes, elevated NLRP3 and ASC expression, activation of caspase‐1, and production of IL‐1β and TNF‐α, and, furthermore, loss of brain‐derived neurotropic factor and postsynaptic density protein‐95 expression in the hippocampus after anesthesia and surgery were abolished by treatment with MCC950.^157^ Nonetheless, supporting evidence still awaits for the role of the NLRP3 inflammasome in coupling activation of the P2X7 receptor to activation of caspase‐1 and production of IL‐1β in PND.

## 4 CNS‐PENETRANT P2X7 RECEPTOR ANTAGONISTS FOR MONITORING AND MITIGATING CNS INFLAMMATION

**Figure 3:**
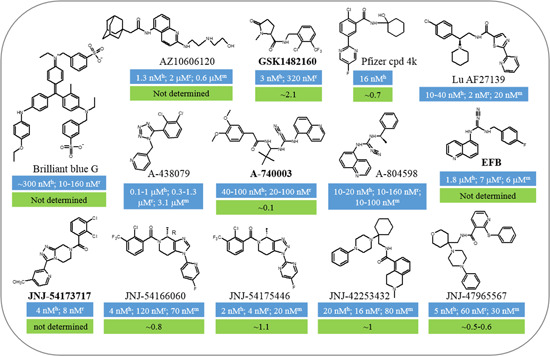
Example P2X7 receptor antagonists and their pharmacological properties. Chemical structures of example P2X7 receptor‐specific antagonists and their potency of inhibiting the human (h), rat (r), or mouse (m) P2X7 receptors, indicated by the IC_50_ (the concentration that inhibits a receptor‐mediated response by 50%) and CNS penetrability, indicated by the brain to plasma (B/P) ratio. Some of them are CNS‐penetrant but the B/P value is not determined. Antagonists highlighted in bold are developed as imaging tracers. See the text for details. CNS, central nervous system. [Color figure can be viewed at wileyonlinelibrary.com]

While earlier in vitro studies demonstrated the P2X7 receptor expression in immune cells, investigation into its role in in vivo settings, particularly in inflammatory diseases, was hindered by the lack of selective antagonists. Studies, using transgenic P2X7 receptor‐deficient mice, provided the first line of definitive evidence to support an important role for the P2X7 receptor in mediating ATP‐induced production of IL‐1β in vivo and the pathogenesis of arthritis.^158^, ^159^ Earlier studies reported that oxATP,^160^ KN‐62 and its structural analog KN‐04,^161^, ^162^ calmidazolium,^163^ and BBG^164^ inhibited endogenous or recombinant P2X7 receptors. Subsequent characterizations indicate that KN‐62 preferentially inhibits the human P2X7 receptor but not the rat P2X7 receptor^165^ and that BBG acts as a P2X7 receptor‐selective antagonist at both human and rodent receptors with a potency of submicromolar concentrations (Figure 3).^166^ BBG has been widely used ever since in in vitro and in vivo studies examining the expression of the P2X7 receptor in a variety of tissues and cells, and its role in physiological and pathological processes, such as traumatic CNS damage discussed above. It is worth emphasizing that caution should be applied in studies using BBG in micromolar concentrations, which can inhibit other P2X receptors^166^ and neuronal voltage‐gated sodium channels.^167^ Studies over the past decades, using pharmacological or genetic intervention in combination with rodent disease models, have collected a large volume of evidence to show activation of the P2X7 receptor as an important mechanism for generation of proinflammatory mediators by immunocompetent cells, and the pathogenesis of numerous inflammatory diseases. These findings raise an interest in targeting the P2X7 receptor to develop anti‐inflammation therapeutics.^36^, ^168^ Considerable efforts from pharmaceutical and biotech companies have been devoted to developing P2X7 receptor‐specific antagonists, mainly through high throughput screening of proprietary chemical libraries followed by structural optimization to improve the physicochemical and pharmacokinetic properties of candidate compounds. As a result, a number and structurally diversity of novel P2X7 receptor antagonists have been discovered.^36^, ^169^, ^170^, ^171^ Figure 3 illustrates a few examples, including AZ10606120,^172^ A‐438079,^173^, ^174^ A‐740003,^175^ A‐804598,^176^ JNJ‐47965567,^177^ JNJ‐42253432,^178^ JNJ‐54166060^179^ and JNJ‐54173717,^180^ GSK1482160,^181^ EFB,^181^ Pfizer compound 4k,^182^ and Lu AF27139.^183^ Readers interested in more information on P2X7 receptor‐specific antagonists can consult reviews.^36^, ^169^, ^171^ It is worth mentioning that AZD9056 from AstraZeneca and CE‐224,535 from Pfizer are the first two P2X7 receptor‐specific antagonists tested in phase 2 clinical trials for the treatment of rheumatoid arthritis, with disappointing outcomes.^184^, ^185^ A placebo‐controlled and double‐blind clinical trial has shown promising use of AZD9056 as an orally active P2X7 receptor antagonist to improve symptoms in adult patients with moderate‐to‐severe Crohn's disease,^186^ which however has been called into question.^187^ More recently, PX7 receptor antagonists have been in clinical testing for inflammatory bowel disease, depression, and other conditions.^188^, ^189^

As mentioned above, recent studies have accumulated strong evidence to indicate an important role of the P2X7 receptor, or more specifically P2X7 receptor‐mediated neuroinflammation, in the pathogenesis of neurodegenerative diseases and psychiatric disorders. These findings trigger an interest in the P2X7 receptor as a therapeutic target for the treatment of these conditions.^6^, ^30^, ^61^, ^64^, ^190^, ^191^ Remarkable medicinal chemistry efforts in the past recent years have led to the discovery of an increasing number of P2X7 receptor‐specific antagonists with great CNS penetrability (Figure 3). P2X7 receptor expression in microglia was upregulated in neurodegenerative diseases and such upregulation bears a causative relationship to neuroinflammation and disease progression. Therefore, there is growing interest in exploring CNS‐penetrant P2X7 receptor antagonists as imaging tracers to monitor brain inflammation using positron emission tomography (PET).^192^, ^193^, ^194^ Indeed, [^11^C]A‐740003,^195^ [^11^C]GSK1482160,^196^, ^197^ [^11^C]JNJ‐54173717,^184^, ^198^ and [^18^F]EFB^199^ (Figure 3) have been tested in both rodent and human brains under healthy and neurodegenerative conditions. We anticipate that development of CNS‐penetrant P2X7 receptor antagonists brings positive impacts in terms of exploring the P2X7 receptor to monitor and mitigate CNS neuroinflammation after traumatic CNS damage. It is clear from the discussion above that the P2X7 receptor expression is upregulated after CNS traumatic damage, which in some cases strongly correlates the severity of neurological and cognitive deficits. CNS‐penetrant P2X7 receptor‐specific antagonists thus can be used as PET imaging tracers to monitor CNS neuroinflammation during the development of secondary injury and related chronic complications. Moreover, CNS‐penetrant P2X7 receptor‐specific antagonists are powerful tools for interrogating the P2X7 receptor mechanisms underlying traumatic CNS damage and, importantly, providing the proof of concept that targeting the P2X7 receptor is a therapeutic strategy to mitigate CNS neuroinflammation, and thereby alleviate CNS damage and improve functional recovery.

## 5 CONCLUDING REMARKS AND PERSPECTIVES

A variety of traumatic CNS damage conditions and related chronic complications discussed in this review continue to be the major sources of morbidity and mortality, with tremendous physical, social, and financial impacts on millions of individuals and families worldwide, representing significant unmet clinical needs. It is clear from the discussion that preclinical studies using rodent models have accumulated compelling evidence to indicate that the ATP‐P2X7 receptor signaling axis plays a vital role in microglial activation and neuroinflammation leading to the loss of neurological and cognitive functions arising from various traumatic CNS damage conditions. Nonetheless, further investigations are required to gain a comprehensive understanding of such signaling mechanisms.

The recent advances in structural biology inform us of the modus operandi of the P2X7 receptor and offer an unprecedented opportunity to develop structure‐guided approaches to discover P2X7 receptor‐specific antagonists. With the increasing availability of CNS‐penetrant P2X7 receptor‐specific antagonists, we anticipate that their use as PET imaging tracers enables both clinical and basic researchers to collect information that helps to gain mechanistic insights, monitor the progression of secondary injury, develop imaging biomarkers, examine drug penetration, occupancy and metabolism, and evaluate therapeutic efficacy. Of course, the ultimate dream is that we can leverage the P2X7 receptor as a therapeutic target to develop effective treatments to limit neuroinflammation, support faster and better recovery from traumatic CNS damage and alleviate related chronic complications.

**Table 1:** Summary of the role of the P2X7 receptor in various traumatic CNS damage conditions and chronic complications.

Conditions	Models	Key observations	References
Spinal cord injury (SCI)	Induced by weight‐drop (contusive)	ATP release and apoptotic cell death occurred in peri‐traumatic regions in rats following SCI. Application of oxidized ATP before SCI, or PPADS shortly after SCI, reduced cell death, and improved motor function recovery.	[90]
	Contusive	Systemic administration of BBG reduced tissue damage and improved motor function recovery after SCI in rats. Treatment with BBG reduced SCI‐induced microglial activation, reactive gliosis, and neutrophil infiltration.	[92]
	Induced by compression (compressive)	P2X7 receptor expression in spinal cord increased in rats after SCI. Intraperitoneal injection of BBG every 12 h for 3 days, starting shortly after SCI, attenuated tissue damage, and improved motor function recovery. Treatment with BBG reduced SCI‐induced upregulation of P2X7 receptor expression in microglia, and upregulation of NLPR3, ASC and caspase‐1 expression, and production of IL‐1β in spinal cord. Treatment with BBG attenuated SCI‐induced blood‐spinal cord barrier dysfunction.	[93]
	Contusive	P2X7 receptor expression in spinal cord microglia increased in rats after SCI. Intraperitoneal injection of A‐438079 attenuated upregulation of P2X7 receptor expression in microglia and upregulation of NLRP3 expression, activation of caspase‐1, and production of IL‐1β in spinal cord.	[103]
	Induced by transient aortic arch occlusion	P2X7 receptor expression in spinal cord increased in mice after SCI. Intrathecal injection of A‐438079, before SCI, prevented activation of caspase‐1 and production of IL‐1β in spinal cord, and suppressed the development of pain hypersensitivity arising from SCI.	[112]
	Induced by bilateral dorsal lesion	Application of BBG suppressed upregulation of P2X7 receptor expression in spinal cord microglia after partial SCI in rats. Treatment with BBG, at the injury site to inhibit activation of spinal cord microglia, prevented SCI‐induced upregulation of the urothelial P2X3 receptor expression and improved SCI‐induced bladder dysfunction.	[102]
Traumatic brain injury (TBI)	Induced by controlled cortical impact (CCI)	Intravenous injection of BBG via tail vein reduced cortical lesion and improved neurological function in mice after TBI. Treatment with BBG shortly after injury attenuated IL‐1β expression. Treatment with BBG, immediately before or shortly or up to 4 h after injury, reduced cerebral edema. P2X7 receptor deficiency reduced IL‐1β expression and cerebral edema.	[124]
	CCI	P2X7 receptor expression in microglia increased in cerebral cortex in rats after TBI. Intravenous injection of BBG via tail vein, after injury, improved neurological function. Treatment with BBG reduced the IL‐1β level and edema in cerebral cortex.	[125]
	CCI	P2X7 receptor expression in microglia increased in cerebral cortex in rats after TBI. Intraperitoneal injection of A‐804598, immediately after injury, improved neurological function. Treatment with A‐804598 reduced upregulation of P2X7 receptor expression in microglia, microglial activation, microglial generation of IL‐1β, and microglial shedding of microvesicle‐like particles. Treatment with A‐804598 reduced apoptotic neuronal cell death and astrogliosis in cerebral cortex.	[126]
Ischemic stroke and ischemia‐reperfusion damage	Ischemia induced by OGD in vitro	Treatment with BBG or removal of extracellular Ca^2+^ attenuated microglial cell death in mouse hippocampal slices during ischemia. P2X7 receptor deficiency reduced ischemia‐induced microglial cell death in mouse hippocampal slices.	[130]
	Ischemia induced by OGD in vitro	Treatment with BBG or removal of extracellular Ca^2+^ suppressed death of rat oligodendrocytes in culture or in oligodendrocytes in mouse and rat optic nerve preparations during ischemia. Treatment with BBG reduced ischemia‐induced myelin damage and loss of white matter function in optic nerve preparations.	[131]
	Ischemia induced by OGD in vitro	**•** Treatment with BBG restored ischemia‐elicited irreversible loss of excitatory synaptic transmission in granule neurons in rat hippocampal slices.	[132]
	Ischemia induced by MCAO	Intraperitoneal injection of BBG every 12 h, starting shortly after transient ischemia, reduced neuronal cell death and infarction, and improved neurological function in rats after transient MCAO and reperfusion. Treatment with BBG reduced neuronal cell death in rat brain slices induced by transient OGD and reperfusion.	[133]
	Ischemia induced by MCAO in vivo or OGD in vitro	Intraperitoneal injection of BBG twice/day, beginning shortly after ischemia, alleviated brain infarction, and improved neurological function in mice after transient MCAO and reperfusion. P2X7 receptor deficiency lessened infarction, neuronal cell death and neurological deficits after transient MCAO and reperfusion. Treatment with BBG or P2X7 receptor deficiency reduced neuronal cell death in mouse cortical slices induced by transient OGD and reperfusion.	[134]
Intracerebral hemorrhage (ICH)	Induced by collagenase injection	P2X7 receptor expression in brain microglia increased in rats after ICH. Intracerebroventricular injection of P2X7‐siRNA, 24 h before ICH, reduced edema and neurological dysfunction. Intraperitoneal injection of BBG daily, starting immediately after ICH, alleviated apoptotic neuronal cell death, hemorrhagic tissue damage, edema and neurological dysfunction. Treatment with P2X7‐siRNA reduced ICH‐induced upregulation of NLRP3 and ASC expression, activation of caspase‐1, and production of IL‐1β. Treatment with BBG reduced ICH‐induced increase in P2X7 receptor, NLRP3 and ASC expression, activation of caspase‐1, and production of IL‐1β, as well as neutrophil infiltration. Treatment with BBG reduced ICH‐induced increase in the expression and activity of NADPH oxidase‐2 and inducible nitric oxide synthase, and formation of peroxynitrite.	[138]
	Induced by autologous blood injection	P2X7 receptor expression in brain, particularly astrocytes and endothelial cells in striatum, was increased in rats after ICH. Intraperitoneal injection of A‐438079 daily for 3 days, starting 30 min after ICH, attenuated neuronal cell death, BBB dysfunction, edema and neurobehavioral function. Intracerebroventricular injection of P2X7‐siRNA before ICH reduced BBB dysfunction, edema and neurological dysfunction. Treatment with A‐438079 or P2X7‐siRNA inhibited activation of RhoA, downregulation of occludin, VE‐cadherin, and ZO‐1 protein expression. Treatment with C3 transferase, 24 h after ICH, improved BBB dysfunction and neurological dysfunction, as well as downregulation of occludin, VE‐cadherin, and ZO‐1 protein expression. Treatment with A‐438079 mitigated BBB dysfunction, neurological dysfunction, downregulation of occluding protein expression, and hemorrhagic tissue damage in rats after collagenase‐injection‐induced ICH.	[140]
	Induced by autologous blood injection	P2X7 receptor expression in brain increased in rats after ICH. Intraperitoneal injection of BBG, shortly after injury, reduced neuronal cell death and edema.	[143]
	Induced by collagenase injection	Treatment with SAM or NaHS reduced apoptotic neuronal cell death, edema and neurological deficits in rats after ICH via downregulating P2X7 receptor expression in microglia. Treatment with SAM or NaHS inhibited LPS/ATP‐induced P2X7 receptor‐mediated upregulation of NLRP3 and ASC expression, activation of NLRP3 inflammasome, and production of IL‐1β in cultured rat microglia.	[141]
	Induced by collagenase injection	P2X7 receptor expression in brain increased in mice after ICH. Intraperitoneal injection of A‐438079, shortly after ICH, prevented increase in expression of P2X7 receptor and NOX2, oxidative stress, apoptotic cell death, edema and neurological dysfunction.	[142]
	Induced by collagenase injection	P2X7 receptor expression in microglia increased in rats after ICH. Perfusion of BBG for 6 h after ICH reduced upregulation of P2X7 receptor expression in microglia, microglial activation, expression of IL‐1β, IL‐6, and TNF‐α. Treatment with BBG, after ICH, reduced astrocyte activation, neuronal loss, and central poststroke pain.	[139]
Subarachnoid hemorrhage (SAH)	Induced by endovascular perforation	Intracerebroventricular administration of P2X7‐siRNA before SAH, or intraperitoneal injection of BBG shortly after SAH, reduced apoptotic neuronal cell death and edema, and ameliorated neurological dysfunction in rats after SAH.	[146, 147]
	Induced by endovascular perforation	Intracerebroventricular administration of P2X7‐siRNA before SAH, or intraperitoneal injection of BBG shortly after SAH, reduced activation of NLRP3 inflammasome and caspase‐1, and production of IL‐1β in rats after SAH.	[147]
Neonatal hypoxia‐ischemia	Hypoxia	P2X7 receptor expression in neocortex and hippocampus increased in mouse pups after hypoxia, and in human neocortex samples from patients with hypoxic/ischemic encephalopathy and seizures. Intraperitoneal injection of A‐438079 or JNJ‐47965567, before hypoxia, reduced neonatal seizures and spiking during hypoxia in mouse pups. Treatment with A‐438079 reduced expression of IL‐1β and precaspase‐1, and activation of caspase‐1.	[149]
Radiation‐induced brain injury (RBI)	Induced by radiation	CSF ATP level was elevated in cerebrospinal fluid from RBI patients and positively correlated with neurological symptoms and edema volume, as well as with CSF level of COX‐2, IL‐6, and TNF‐α. P2X7 receptor expression in microglia increased in irradiated mice. Intraperitoneal injection of BBG for 7 consecutive days, starting 2 h before irradiation, preserved spatial memory, and reduced neurological deficits in irradiated mice. Treatment with BBG prevented radiation‐induced increase in P2X7 receptor expression, microglial activation, expression of COX‐2, IL‐6, and TNF‐α, and neurogenesis in hippocampus. Treatment with BBG or P2X7‐siRNA reduced radiation‐induced microglial activation, microglial expression of COX‐2, IL‐6, and TNF‐α, and apoptosis of neuronal cells cocultured with microglia.	[152]
Perioperative neurocognitive disorder (PND)	Induced by carotid arterial surgery under anesthesia	P2X7 receptor expression in microglia increased in mice after PND. Intraperitoneal injection of BBG daily for a week, starting 15 min before surgery, reduced cognitive dysfunction. P2X7 receptor deficiency abolished surgery‐induced cognitive dysfunction Treatment with BBG reduced surgery‐induced microglial activation, expression of precaspase‐1, activation of caspase‐1, and generation of IL‐1β in hippocampus. P2X7 receptor deficiency prevented surgery‐induced generation of IL‐1β.	[156]

Abbreviations: ASC, apoptosis‐associated speck‐like protein containing a caspase activation and recruitment domain; ATP, adenosine 5ʹ‐triphosphate; BBB, blood‐brain barrier; BBG, Brilliant Blue G; COX‐2, cyclooxygenase‐2; CSF, cerebrospinal fluid; IL, interleukin; LPS, lipopolysaccharide; MCAO, transient middle carotid artery occlusion; NaHS, sodium hydrosulphide; NLPR3, NOD‐, leucine‐rich repeat‐ and pyrin domain‐containing protein 3; OGD, oxygen and glucose deprivation; PPADS, pyridoxal‐5′‐phosphate‐6‐azophenyl‐2′,4′‐disulphonic acid; SAM, S‐adenosyl‐l‐methionine; siRNA; small interference RNA; TNF, tumor necrosis factor; VE, vascular endothelial; ZO‐1, zonula occluden‐1.

## CONFLICT OF INTEREST STATEMENT

All authors declare no conflict of interest.

## Supporting information

Supporting information.

## Data Availability

Data sharing is not applicable to this article as no new data were created or analyzed in this study.
