# Supplementary material for: Leveraging the ATP‐P2X7 receptor signalling axis to alleviate traumatic CNS damage and related complications
Source: Med Res Rev. 2023 Mar 16;43(5):1346–73. doi: 10.1002/med.21952 (PMC10947395; doi:10.1002/med.21952)
Supplement: Supplementary file 1 — Supporting information. [file MED-43-1346-s001.pdf]

WILEY

Medicinal Research Reviews

Proposal and pre-submission email communications with Dr A E Hargrove, Editor-in-Chief, MRR

Dear Prof. Jiang,

Thank you for your email. Out of respect to our authors, we try to prevent overlap in reviews in MRR, which is why the initial decision was to decline your proposal. It was not a question of expertise or appropriateness of topic in this case.

Based on your email, however, I consulted again with the editorial team, and we have decided that there is enough difference between the existing manuscript and your proposal to allow both. Combined with your expertise in the subject, we agree that this could be a significant contribution to the field and are happy to process it for peer review.

When you are ready, please double check the Author Guidelines:
https://onlinelibrary.wiley.com/page/journal/10981128/homepage/forauthors.html

and submit through the Scholar One portal:
https://mc.manuscriptcentral.com/med

Thank you again for your interest in contributing to *Medicinal Research Reviews!*

All the best,

Amanda

==========================
Amanda E. Hargrove, Ph.D. (she/hers)
Associate Professor of Chemistry
3219 French Family Science Center
124 Science Drive, Box 90346
Duke University
Durham, NC 27708-0346
919-660-1522
https://sites.duke.edu/hargrovelab/
Twitter @hargrovelab

Editor-in-Chief, Medicinal Research Reviews
https://onlinelibrary.wiley.com/journal/10981128

From: Lin-Hua Jiang <L.H.Jiang@leeds.ac.uk>
Sent: Monday, November 8, 2021 12:11 PM
To: Amanda E. Hargrove <amanda.hargrove@duke.edu>
Cc: mrreditorial@wiley.com <mrreditorial@wiley.com>
Subject: RE: Your proposal entitled "Leveraging the role of the P2X7 receptor in neuroinflammation to mitigate traumatic CNS damage and support functional recoveryhas been submitted to Medicinal Research Reviews

WILEY
Medicinal Research Reviews

Dear Prof A Hargrove,

Many thanks for your kind consideration of our proposal of writing a review manuscript for MRR.

The P2X7 receptor is an important molecular mechanism mediating inflammation of tissues including the CNS and, not surprisingly, it has been identified as an attractive anti-inflammation target. MRR has recently published a review on this interesting topic, focusing on in neuroinflammation in neurodegenerative diseases (Calzaferri F, Ruiz-Ruiz C, de Diego AMG, de Pascual R, Méndez-López I, Cano-Abad MF, Maneu V, de Los Ríos C, Gandía L, García AG (2020) The purinergic P2X7 receptor as a potential drug target to combat neuroinflammation in neurodegenerative diseases, Med Res Rev, 40(6):2427-2465). This review have omitted by large the recent groundbreaking works (e.g., McCarthy AE, Yoshioka C, Mansoor SE (2019) Full-Length P2X(7) Structures Reveal How Palmitoylation Prevents Channel Desensitization, Cell, 179(3):659-670.e13), which inform the structural basis for the modus operandi of the P2X7 receptor. This paper has not touched on the recent progress in revealing the critical role of P2X7-mediated neuroinflammation in multiple traumatic CNS damage, which are, albeit less prevalent, equally or arguably more debilitating than neurodegenerative diseases, and the promising perspective on the use of CNS-penetrant P2X7 antagonists as a therapeutic strategy for such conditions. The lack of the recent developments on these important forefronts has compelled us to propose writing this review manuscript. As a reader and as a reviewer of MRR, I have come across quite a few papers including the above one on the P2X7 receptor by authors with a pretty thin track record on the research topic they are writing. We strongly believe that we are well or better qualified for the proposed review.

Thus, while we highly respect your decision, we find it a bit intriguing to hear that our proposal deems not fit the publication criteria.

Kind regards,

Lin-Hua Jiang

Lin-Hua Jiang, PhD
School of Biomedical Sciences, Faculty of Biological Sciences, Rm6.44E Garstang Building, University of Leeds, Leeds LS2 9JT, UK
Email: l.h.jiang@leeds.ac.uk; Telephone: 0113 3434231;
Orcid ID: 0000-0001-6398-0411; Scopus ID: 35200765000;
Webpage: https://biologicalsciences.leeds.ac.uk/biomedical-sciences/staff/92/dr-lin-hua-jiang

From: Amanda E. Hargrove [mailto:amanda.hargrove@duke.edu]
Sent: 08 November 2021 14:39
To: Lin-Hua Jiang <L.H.Jiang@leeds.ac.uk>
Cc: mrreditorial@wiley.com
Subject: Re: Your proposal entitled "Leveraging the role of the P2X7 receptor in neuroinflammation to mitigate traumatic CNS damage and support functional recoveryhas been submitted to Medicinal Research Reviews

Dear Dr. Jiang,

I hope you are well and thank you for your interest in publishing with Medicinal Research Reviews.

WILEY
Medicinal Research Reviews

We have conducted an editorial evaluation and concluded that the proposed manuscript does not fit our publication plan. Because we receive far more manuscripts than we can possibly publish, we subject all manuscripts to an initial screening by considering many factors including timeliness of the manuscript, the level of activity of the field, author's level of expertise in the field as demonstrated by prior publications on the same subject, whether there have been recent reviews on the same subject as well as the need for the journal to publish manuscripts from diverse areas and diverse regions.

We wish you the very best in finding an appropriate journal for your interesting work and hope that you will consider Medicinal Research Reviews for future submissions.

Kind regards,

Prof. Amanda Hargrove, Ph.D.
Editor-in-Chief
Medicinal Research Reviews

From: Microsoft Power Apps and Power Automate <microsoft@powerapps.com>
Sent: Saturday, October 2, 2021 10:36 AM
To: l.h.jiang@leeds.ac.uk <l.h.jiang@leeds.ac.uk>
Cc: mrreditorial@wiley.com <mrreditorial@wiley.com>
Subject: Your proposal entitled "Leveraging the role of the P2X7 receptor in neuroinflammation to mitigate traumatic CNS damage and support functional recoveryhas been submitted to Medicinal Research Reviews

A new proposal has been submitted via Microsoft Forms from l.h.jiang@leeds.ac.uk.
Proposal title: "Leveraging the role of the P2X7 receptor in neuroinflammation to mitigate traumatic CNS damage and support functional recovery"
Article type: "Minireview Article"
Corresponding author(s):"Lin-Hua Jiang"
Corresponding author(s) affiliation(s):School of Biomedical Sciences, University of Leeds
Outline: 1. Introduction 2. P2X7 receptor architecture and structural basis for ATP binding and receptor activation 3. Role of the P2X7 receptor in traumatic CNS damage and mechanisms for neuroinflammation 3.1 Spinal cord injury 3.2 Ischemic and haemorrhagic stroke 3.3 Hypoxia-induced neonatal seizures 3.4 Traumatic brain injury 3.5 Radiation-induced brain injury 3.6 Postoperative cognitive dysfunction 4. CNS-penetrant P2X7 receptor antagonists for imaging neuroinflammation and therapeutics 5. Concluding remarks and perspectives

WILEY
Medicinal Research Reviews
